# The Inositol-5-Phosphatase SHIP1: Expression, Regulation and Role in Acute Lymphoblastic Leukemia

**DOI:** 10.3390/ijms26146935

**Published:** 2025-07-19

**Authors:** Patrick Ehm, Manfred Jücker

**Affiliations:** Institute of Biochemistry and Signal Transduction, Center for Experimental Medicine, University Medical Center Hamburg-Eppendorf, Martinistr. 52, 20246 Hamburg, Germany; p.trickehm@gmail.com

**Keywords:** SHIP1, inositol 5-phosphatase, PI3K/AKT-signaling, acute lymphoblastic leukemia

## Abstract

Despite the successes achieved in recent years in the treatment of childhood acute lymphoblastic leukemia (ALL), high-risk ALL in particular still represents a considerable challenge, with poorer outcomes. The PI3K/AKT/mTOR signaling pathway is frequently constitutively activated in ALL and consequently leads to unrestricted cell proliferation, without showing frequent mutations in the most important representatives of the signaling pathway. Recent studies have shown that fine balanced protein expression is a common way to adjust oncogenic B cell directed receptor signaling and to mediate malignant cell proliferation and survival in leukemic cells. Too low expression of inhibitory phosphatases can lead to constitutive signaling of kinases, which are important for cell proliferation and survival. In contrast, marked high expression levels of key phosphatases enable cells with distinct pronounced oncogenic B cell directed receptor signaling to escape negative selection by attenuating signal strength and thus raising the threshold for deletion checkpoint activation. One of the most important B cell receptor-dependent signaling cascades is the PI3K/AKT signaling pathway, with its important antagonist SHIP1. However, recent data show that the inositol-5-phosphatase SHIP1 is differentially expressed across the heterogeneity of the ALL subtypes, making the overall therapeutic strategy targeting SHIP1 more complex. The aim of this article is therefore to provide an overview of the current knowledge about SHIP1, its expression in the various subtypes of ALL, its regulation, and the molecules that influence its gene and protein expression, to better understand its role in the pathogenesis of leukemia and other human cancers.

## 1. Introduction

Drugs are intended to target a particular vulnerability in a tumor, generated in most cases by its dependence on an oncogene and/or loss of a tumor suppressor. Deregulated kinases are frequently found to be oncogenic, so phosphorylation reactions play an important role in the regulation of protein activities, as well as in numerous signal transduction processes, and are therefore a critical mechanism of the cell. There are several ways for kinases to become involved in cancers, e.g., aberrant phosphorylation, mutations, and chromosomal translocations, as well as mis-regulated expression. Oncogenic tyrosine kinases can mimic survival and proliferation signals from a constitutively active pre-BCR (B cell receptor) in B cell leukemia [1,2]. Phosphatases are important antagonists of kinases and try to balance the activation state of the respective signaling pathway in an, at least in part, complex regulatory system that is not yet fully understood. In addition to proteins, inositols and phosphatidylinositols can also serve as signaling molecules and can be modified by phosphorylation, partly by the well-known phosphoinositide 3-kinase (PI3K).

The PI3K/AKT/mTOR (phosphoinositide 3-kinase/protein kinase B/mechanistic target of rapamycin) signaling pathway was found to be constitutively activated in various solid cancers and the majority of hematological malignancies, including ALL [3]. The kinase AKT is an essential downstream target of PI3K and, together with MTOR, has a significant influence on the proliferation, survival, and resistance development of ALL cells [4,5]. The importance of the PI3K/AKT/mTOR signaling pathway is highlighted by the fact that PIK3CA (catalytic subunit alpha) and PTEN (phosphatase and tensin homolog) have been identified as two of the most frequently mutated genes in cancer [6,7]. In contrast to other types of cancer, very few mutations are found in the main components of the PI3K/AKT/mTOR signaling pathway in acute leukemias [8]. This raises the question of how the AKT signaling pathway becomes constitutively activated in a frequent number of ALL cases, because constitutive activation of AKT can be detected in 87% of patients with T-ALL and in over 80% of patients with B-ALL [9,10,11]. After recruitment to ITIM-bearing inhibitory receptors (immunoreceptor tyrosine-based activation motif), hematopoietic phosphatases like SHIP1 (src homology 2 domain-containing inositol phosphatase), an important antagonist of the AKT signaling pathway, counteract activated BCR signaling in hematopoietic cells or its oncogenic mimicry [12].

Due to numerous indications in recent years that SHIP1 is an important negative regulator of the PI3K/AKT/mTOR signaling pathway and BCR-dependent signaling, we take a closer look at its tumor suppression, its expression in the various subtypes of ALL, its regulation, and its role in the progression of ALL, and summarize these areas in this review article.

## 2. The PI3K/AKT Signaling Pathway

**Figure 1:**
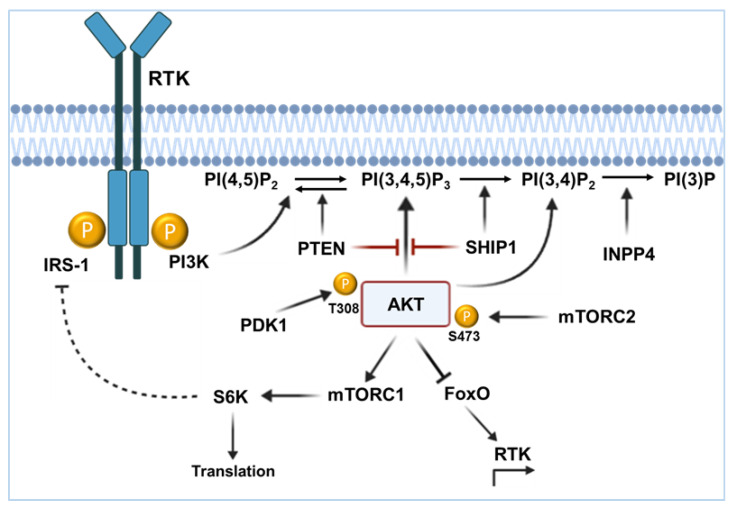
**The PI3K/AKT signaling pathway.** Upon stimulation of receptors with intrinsic or associated tyrosine kinase activity (RTK), the PI3 kinase (PI3K) is recruited to the plasma membrane. PI3K phosphorylates its substrate PI(4,5)P_2_ to PI(3,4,5)P_3_, whereupon the serine/threonine kinase AKT binds to the membrane with its PH domain. AKT is phosphorylated by PDK1 at threonine residue 308 and by mTORC2 at serine residue 473. The then activated AKT phosphorylates more than 100 substrates and subsequently regulates numerous cell functions, such as proliferation, protein synthesis, and survival. The phosphatases PTEN and SHIP1 act as antagonists of the signaling pathway. PTEN dephosphorylates PI(3,4,5)P_3_ to PI(4,5)P_2_ and SHIP1 dephosphorylates PI(3,4,5)P_3_ to PI(3,4)P_2_. AKT can be recruited to both PtdIns(3,4,5)P_3_ and PI(3,4)P_2_. PI(3,4)P_2_ is dephosphorylated to PI(3)P by INPP4B.

The PI3K/AKT signaling pathway plays an important role in cell growth, differentiation, survival, and apoptosis. After binding of ligands to the receptor tyrosine kinase, PI3K is recruited to the plasma membrane (Figure 1). The interaction with the phosphotyrosine residues of the receptor occurs via the SH2 domain (src homology 2 domain) of the p85 subunit of the PI3K. Phosphatidylinositol 4,5-bisphosphate (PtdIns(4,5)P_2_) represents an important substrate of the PI3K and is anchored in the lipid layer of the membrane via two fatty acids. Due to the binding of the PI3K to the phosphotyrosine of the receptor, a conformational change and activation of the catalytic p110 subunit of the PI3K occurs [13]. The p110 subunit then converts the PtdIns(4,5)P_2_ to PtdIns(3,4,5)P_3_. The recruitment and binding of signaling proteins with lipid-binding PH (pleckstrin homology) domains then occurs for the formed PtdIns(3,4,5)P_3_. A protein that can bind to PtdIns(3,4,5)P_3_ with its N-terminal PH domain is the serine/threonine kinase AKT. Binding of AKT to the membrane causes a conformational change, whereby AKT is phosphorylated by PDK1 (phosphoinositide-dependent protein kinase-1) at threonine residue 308 and mTORC2 (mechanistic target of rapamycin complex 2) at serine residue 473 and thus becomes activated. Through activation, AKT is able to phosphorylate and activate numerous other substrates. AKT is attributed several properties that are important for the development of malignant diseases. For example, AKT promotes cell survival through phosphorylation and inhibition of Bad, promotes proliferation by phosphorylation and thus inactivation of glycogen synthase kinase 3β (GSK3β), and promotes protein synthesis by activation of mTORC1 (mammalian target of rapamycin, complex 1) [14]. However, the activation of AKT can be prevented by the phosphatases PTEN and SHIP1. PTEN hydrolyzes the reverse reaction of PtdIns(3,4,5)P_3_ to PtdIns(4,5)P_2_, whereas SHIP1 hydrolyzes the reaction of PtdIns(3,4,5)P_3_ to PtdIns(3,4)P_2_.

PI(3,4,5)P_3_ enables PH domain proteins such as PDK1, AKT, and TEC (tec protein tyrosine kinase) kinases to be recruited to the membrane. PH domain adapter proteins (GABs, SKAPs, Bam32, and TAPP) can also bind to the phosphoinositides [15]. Recent findings indicate that in some cells AKT can be recruited more efficiently to PI(3,4)P_2_ than to PI(3,4,5)P_3_ [16,17]. In particular, in the absence of adapter proteins such as TAPP, PI(3,4)P_2_ is available to promote AKT recruitment [18,19]. PI(3,4)P_2_ is dephosphorylated by INPP4B to PI(3)P [20,21]. It is possible that both PI(3,4,5)P_3_ and PI(3,4)P_2_ are important for the complete activation of AKT. PLCγ (phospholipase C gamma) subsequently converts PI(4,5)P_2_ to DAG (diacylglycerol) and I(1,4,5)P_3_. If not attenuated, constitutive activation of the AKT signaling pathway negatively influences the response to therapeutic treatments, correlates with drug resistance, and is associated with a poor prognosis for ALL patients [22,23,24,25,26].

### 2.1. Enzymatic Activity of SHIP1

The regulatory function of SHIP1 is characterized by the conversion of phosphatidylinositol-3,4,5-trisphosphate (PtdIns(3,4,5)P_3_) to phosphatidylinositol-3,4-bisphosphate (PtdIns(3,4)P_2_) [27]. This involves relocalization from the cytoplasm, where SHIP1 is predominantly located, to the cell membrane. In this case, SHIP1 inhibits the activation of AKT via the degradation of PtdIns(3,4,5)P_3_ and thus influences the differentiation and proliferation of hematopoietic cells. Furthermore, the inositol phosphate metabolism is regulated by SHIP1 through conversion of inositol-1,3,4,5-tetrakisphosphate (Ins(1,3,4,5)P_4_) to inositol-1,3,4-trisphosphate (Ins(1,3,4)P_3_) [27].

### 2.2. The B Cell Activation

**Figure 2:**
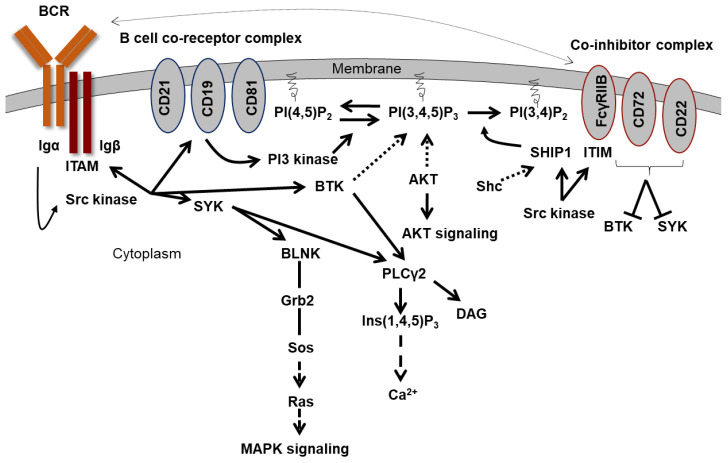
**Schematic representation of B cell activation and recruitment of SHIP1 to the membrane.** Antigen binding induces activation of the B cell receptor complex. Non-receptor tyrosine kinases of the Src family are associated with the cytoplasmic part of the receptor complex and are activated upon ligand binding. The SRC kinase, in turn, activates SYK, BTK, and PI3K via CD19. SYK subsequently activates the MAPK signaling pathway and, together with BTK, promotes calcium efflux. PI3K generates PI(3,4,5)P3, thus activating the AKT signaling pathway. The B cell response can be inhibited by phosphorylation of the ITIM motif of the inhibitory Fcγ receptor IIB after SHIP1 recruitment.

In B cells, the binding of an antigen induces the activation of the B cell receptor complex (BCR complex). The B cell receptor complex is composed of a membrane-bound immunoglobulin (IgD and IgM in mature naive B cells, IgA, IgG or IgE in activated B cells) and a heterodimer of Igα (CD79α) and Igβ (CD79β). Non-receptor tyrosine kinases of the Src family such as LYN or FYN are associated with the cytoplasmic portion of the receptor complex and are activated after ligand binding. They phosphorylate the ITAMs of Igα and Igβ (Figure 2). The phosphotyrosines then serve as binding sites for proteins with SH2 domains or phosphotyrosine binding domains (PTB). A large number of proteins have such domains, including kinases, phosphatases, adapter proteins, and transcription factors [28,29]. LYN also phosphorylates and activates SYK (spleen associated tyrosine kinase) and the TEC kinase BTK (Bruton’s tyrosine kinase) [30,31]. SYK can then bind to the phosphorylated ITAMs with its two SH2 domains and phosphorylate the adapter protein BLNK (B cell linker protein), which is also located on the membrane [32,33]. GRB2 (growth factor receptor-bound 2) then binds to the phosphorylated BLNK with the bound SOS (son of sevenless). SOS activates RAS (rat sarcoma) and thus the MAP kinase (mitogen-activated protein) signaling pathway [34,35]. PLCγ2 becomes activated via SYK and BTK and cleaves PtdIns(4,5)P_2_ to DAG and Ins(1,4,5)P_3_ [30]. This ultimately causes Ca^2+^ to flow out of intracellular stores.

The so-called complement system is also part of B cell activation. The antigen is bound by the complement fragment C3d. The fragment can bind to the type 2 complement receptor (CR2, CD21) on B cells. Together with CD19 and CD81, it forms part of the B cell co-receptor complex. Starting from the ITAMs of Igα and Igβ, LYN phosphorylates an ITAM in the cytoplasmic part of CD19 [36]. This leads to the binding and activation of the PI3 kinase, which generates PtdIns(3,4,5)P_3_, as described before, and thus initiates the AKT signaling pathway. In contrast, an antibody feedback process is initiated to regulate the immune response. The antibody production of an activated B cell is inhibited by secreted IgG. The secreted IgG antibodies form complexes with the antigen and bind with their Fc portion to the inhibitory Fcγ receptor IIB (CD32). The antigen-mediated interaction between the BCR and the inhibitory Fcγ receptor IIB leads to tyrosine phosphorylation of the ITIM in the cytoplasmic part of the Fcγ receptor IIB by the BCR-associated Src kinase LYN [37]. This leads to the binding of SHIP1 via its SH2 domain to the ITIM. SHIP1 dephosphorylates PtdIns(3,4,5)P_3_ to PtdIns(3,4)P_2_, whereby BTK and AKT can no longer be actively recruited to the membrane and are inhibited. Thus, SHIP1 regulates immune response in B cells.

### 2.3. Recruitment of SHIP1 to the Membrane

Several mechanisms have been described by which SHIP1 can reach the plasma membrane to dephosphorylate PtdIns(3,4,5)P_3_. This process depends on the cell type and the available components, such as receptors and interaction partners. Accordingly, recruitment to the membrane can occur directly via binding to a receptor (ITIM) or indirectly via binding to adapter proteins such as SHC (src homologous and collagen) or DOKs (downstream of kinase). For example, after activation of the inhibitory receptor Fcγ receptor IIB, SHIP1 binds with its SH2 domain to the cytoplasmic phospho-tyrosine in the ITIM motif of the receptor [38,39]. After recruitment to the membrane, tyrosine phosphorylation of SHIP1 occurs by membrane-associated tyrosine kinases from the Src kinase or Janus kinase family [40,41]. The tyrosine-phosphorylated NPXY motifs can subsequently serve as a docking site for proteins with PTB domains (phosphotyrosine-binding domains) such as SHC and DOK1 [42]. After IL-3 stimulation, JAK2 (janus kinase 2) phosphorylates the cytoplasmic part of the beta subunit of the IL-3 receptor, allowing SHC to bind to the receptor with its PTB domain. SHC becomes tyrosine-phosphorylated by LYN or JAK2, which in turn allows SHIP1 with its SH2 domain to bind to SHC and thus reach the membrane [43,44]. By binding to SHC, SHIP1 also blocks the interaction of SHC and the GRB2–SOS complex, thereby preventing RAS activation [45,46,47]. Subsequently, SHIP1 can also be tyrosine-phosphorylated by LYN or JAK2 [40,41,43].

In another mechanism, GRB2 can bind to the tyrosine-phosphorylated receptor or to SHC, which is tyrosine-phosphorylated by LYN and SYK. SHIP1 then binds to the two SH3 domains of GRB2 at the membrane.

## 3. The Human Inositol-5-Phosphatase (SHIP1) and Its Structure

The human SH2-domain-containing inositol-5-phosphatase (SHIP1) belongs to the family of inositol-5-phosphatases and was identified as a protein of 1188 amino acids [48] and 1189 amino acids [49]. In the latter form, an additional valine at position 117 is described, which, however, is not associated with any phenotype. Therefore, we use the delta-valine form for further declaration.

**Figure 3:**
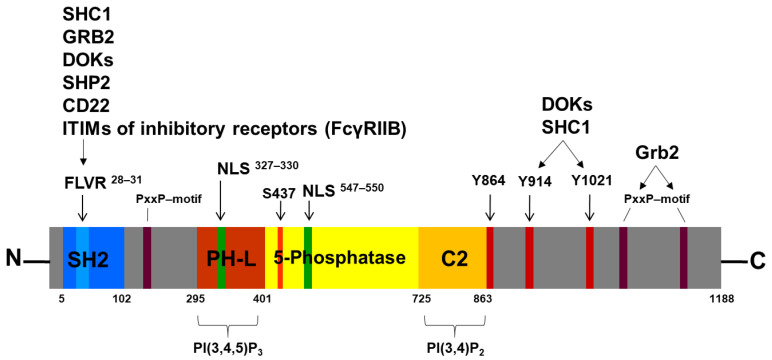
**Schematic representation of the structure of SHIP1.** The SH2 domain (blue) is located at the N-terminus of SHIP1 and contains the FLVR motif as a binding site for various proteins, particularly receptors with ITIM. The characteristic 5-phosphatase domain of SHIP1 with PH-L and C2 domains is located centrally (yellow). In addition, SHIP1 has two nuclear localization signals (green), two NPXY motifs (Y914 and Y1021; red), and several PxxP motifs (purple). The serine residue 437 (orange) is responsible for regulating phosphatase activity. The significance of the tyrosine residue 864 (red), located adjacent to the C2 domain, is still unknown.

SHIP1 is encoded by the INPP5D gene. Human SHIP1 was cloned for the first time in 1996 [48] and has an apparent molecular weight of 145 kDa. In addition, other molecular weights of SHIP1 have been described, which can arise through alternative splicing (135 kDa) or protein degradation. For example, a truncated p102 form can arise by proteolytic cleavage at the C-terminus by a PMSF-inhibitable serine protease during cell lysis in vitro [50]. The most important domains and motifs of SHIP1 are the SH2 domain with its FLVR motif, the 5-phosphatase domain, and the NPXY and PxxP motifs (Figure 3).

### 3.1. The SH2 Domain and the FLVR Motif

SH2 domain-containing proteins are important signal transduction molecules within the cell, and the SH2 domain is responsible for cellular localization, substrate recruitment, and the regulation of kinase activity (e.g., Src kinase family or FES) [51,52]. SHIP1 possesses an N-terminal SH2 domain (AA 5-102), for which several mutations have been identified affecting the FLVR motif (AA 28-31) or located in close proximity to the FLVR motif of SHIP1 (e.g., F28L, L29F, and S33T) [53]. Reduced binding to tyrosine-phosphorylated proteins has previously been described for the F28L mutation, but the arginine in the FLVR motif is the only amino acid directly involved in phosphotyrosine binding [54,55].

Analysis of patient-derived SHIP1-FLVR motif mutations showed reduced protein expression and increased phospho-AKT S473 levels [56]. Amino acids with aromatic residues at position 28 of SHIP1 preserved protein stability (F28Y and F28W), while replacement with non-aromatic amino acids severely compromised protein stability (F28L, F28I, F28V, and F28A), thus affecting signal transduction and cell growth [56]. A detailed structural analysis revealed that F28 forms hydrophobic surface contacts, particularly with W5, I83, L97, and P100, which could be maintained by tyrosine and tryptophan residues, but not by non-aromatic residues at position 28 [56]. Further SH2 domain mutations of various signaling proteins (FER, ZAP-70, JAK3, ITK, and BTK) can also lead to their destabilization [56,57,58,59]. Analysis of the extended G(S/T)FLVR(E/D)S motif [60] using the phosphosite database showed that several putative phosphorylation sites (S27, S33, S35, Y40, and Y46) are located in close proximity to the SHIP1-FLVR motif, some of which are highly conserved among SH2-containing proteins [61]. Interestingly, mutations of the putative phosphorylation sites S27 and S33 adjacent to the SHIP1-FLVR motif also have an impact on its protein stability [56]. These results further support a functional role of SHIP1 as a tumor suppressor protein and indicate a regulation of protein expression of SH2 domain-containing proteins via the FLVR motif. Phosphorylation of specific amino acids of proteins can initiate protein degradation or mask a degradation signal [62].

### 3.2. The 5-Phosphatase Domain

SHIP1 has a central 5′-phosphatase domain (AA 397–863), which is responsible for its catalytic activity. This domain contains two highly conserved inositol-5-phosphatase motifs (AA 582–593 and AA 663–679) [48]. A C2 domain (AA 725–863) was also identified within the phosphatase domain. The C2 domain of SHIP1 binds to PI(3,4)P_2_ and activates SHIP1 allosterically [63,64]. The PH-like domain (or PH-related domain) with the amino acid residues K370 and K397 can mediate the binding of SHIP1 to the membrane-bound PIP3 [65].

Interestingly, a number of mutations were identified within the conserved phosphatase domain of SHIP1 (e.g., G585K, R673Q, E452K, and R551Q), which affected enzymatic activity and, to some extent, protein expression (G585K and R673Q) [66].

Of particular interest could be the putative phosphorylation site on tyrosine residue 864 of SHIP1, which is located c-terminal to the C2 domain (AA 725–863). Interestingly, tyrosine residue 864 is the putative phosphorylation site of SHIP1 with the most hits in the phosphosite database [61], but no functional information has currently been published for this site [67]. Presumably, the phosphorylation of SHIP1 at tyrosine residue 864 could change the conformation of the protein and thus sterically hinder the binding of the substrate to the active center. The phosphorylation site S440 (human: S437), which is located in the phosphatase domain, is phosphorylated by PKA and has already been identified as an allosteric activation site [68]. Interestingly, allosteric activators (AQX-MN100 and AQX-1125) have already been developed that bind to the C2 domain [69,70]. In addition, the SHIP1 activator AQX-435 shows preclinical efficiency in malignant B cells [71]. The C2 domain of SHIP1 normally binds to the endogenous PI(3,4)P_2_ and thus activates SHIP1 allosterically. A SHIP1 mutation that shows constitutively increased activity could possibly promote the survival of malignant B cells in the context of clonal B cell selection. The R620W mutation of the phosphatase PTPN22, which acts as a negative regulator of SYK, leads to an increase in its phosphatase function [72]. This enhancement subsequently leads to an inhibition of BCR signaling strength, which helps to avoid negative selection of the cell.

### 3.3. The NPXY and PxxP Motifs

Two NPXY motifs are located at the C-terminus. One represents an NPNY motif (AA 911–914) and the other is an NPLY motif (AA 1018–1021). After tyrosine phosphorylation of these motifs, an interaction with phosphotyrosine binding domains (PTB domains) of other proteins is possible. Depending on the three residues located C-terminal to the tyrosine residue in the NPXY motif, these can also represent binding sites for other SH2 domains [73]. The tyrosine residue 1021 of SHIP1 is characterized as a phosphorylation site, which plays a role in the stability of membrane binding via interaction partners, the initiation of proteasomal degradation, and dimerization with the SH2 domain of SHIP1 [73,74].

Via the SH2 domain and the NPXY motifs, SHIP1 is able to interact with adapter proteins such as SHC, GABs (GRB2-associated-binding protein 1/2) GRB2, various DOKs (downstream of kinase 1/2/3), the protein tyrosine phosphatase SHP2, the Src kinase family member LYN, CD22, and the ITIMs (immunoreceptor tyrosine-based-inhibitory motifs) of inhibitory receptors [67,75,76]. In addition, the C-terminus of SHIP1 is very proline-rich and has several PxxP motifs that can act as binding sites for SH3 domains.

### 3.4. The Nuclear Localization Signals (NLS) and Nuclear Export Signals

Two nuclear localization signals (NLS) and five nuclear export signals (NES) have been described [77,78]. Nuclear import is mediated by the N-terminal K^327^KSK and the K^547^KLR located in the phosphatase domain. Both signals correspond to the consensus motif for canonical monopartite nuclear localization signals [77]. Interestingly, four SHIP1 mutants (K210R, N508D, V684E, and Q1153L) derived from AML patients showed a nuclear accumulation after expression in AML cells [78]. In addition, the mutation N508D caused an increased proliferation of AML cells in comparison to the wild-type SHIP1. These data suggested that enhanced accumulation of SHIP1 mutants in the nucleus may be a contributory factor to abnormally high proliferation of leukemic cells. In addition, SHIP1 colocalizes in nucleolar cavities with p53 and components of PML nuclear bodies (e.g., SP100, SUMO-1, and CK2) [79]. Treatment of cells with the proteasome inhibitor MG132 causes an enlargement of nucleolar SHIP1-containing structures. These results indicate that nonphysiologically high protein levels can alter the intracellular targeting of proteins, which can contribute to several diseases [80].

Four of the five nuclear export signals are localized in the phosphatase domain (I^481^HTLWNIRI, I^574^THRFTHLFWF, F^584^GDLNYRVDL, and L^842^TGHFQGEIKL). The fifth NES is located C-terminal to the phosphatase domain (V^917^GPFGPPMPL) [78].

## 4. The SHIP1 Mutation Status

The activation of the PI3K/AKT signaling pathway plays an important role in the development and disease progression of many types of cancer. In the various tumor entities, the PI3K/AKT signaling pathway is more frequently affected by genetic changes than any other cell signaling pathway. In addition to the loss of PTEN, mutations in the catalytic p110α subunit of PI3K, in AKT, and in receptor tyrosine kinases within this signaling pathway have been described [81,82,83]. Therefore, numerous clinical studies have aimed to inhibit the PI3K/AKT/mTOR signaling pathway. As a result, PI3K/AKT/mTOR-targeted therapies are being promoted for many types of cancer [84,85,86,87,88,89]. Capivasertib, a selective AKT inhibitor, with fulvestrant was approved for patients with ER-positive and HER2-negative breast cancer whose tumors harbored PIK3CA/AKT1/PTEN alterations [90]. However, comparatively few clinical trials are being conducted to inhibit this signaling pathway in acute childhood lymphatic leukemia. The ClinicalTrials.gov database contains over a hundred entries for clinical trials with PI3K/AKT/mTOR inhibitors in renal cell carcinoma or breast cancer. However, there are just 19 entries for the corresponding treatment of acute lymphoblastic leukemia (https://clinicaltrials.gov/, accessed on 16 April 2025). One reason for that could be that mutations of the INPP5D gene (SHIP1) in hematopoietic cells (126 mutations in 8402 samples examined; 1.5% of cases) are rare [53]. Five mutations of the INPP5D gene in patients with acute lymphoblastic B-cell leukemia were identified in the COSMIC database. However, ALL patients with a SHIP1-ABL1 fusion protein were described [91,92]. Here, the translocation links the 5′-part of the INPP5D gene (exon 1–9; including SH2 domain) to the 3′-part of the ABL1 gene (from exon 2). In consequence, the fusion protein could act as an activated tyrosine kinase and affect normal SHIP1 function in a dominant-negative manner [91]. In addition, an INPP5D-ATG16L1 fusion transcript was observed [93]. A detailed analysis of the dataset from Chouvarine and coworkers [94] showed that several SHIP1 fusion proteins were found. In detail, these fusion proteins were frequently identified in the Ph-like subtype [11]. Interestingly, not only is the mutation status of SHIP1 very low in B-ALL cases, but the other components of the PI3K/AKT/mTOR signaling pathway are also much less frequently affected by mutations compared to other types of leukemia or carcinomas. In accordance with this fact, it is striking that inactivating PTEN mutations occurred in more than 20% of patients with T-cell leukemia (163 of 805 patients), but no mutations or deletions were found in 925 B-cell leukemia samples examined [95]. For this purpose, data from the COSMIC database [53] are summarized in Table 1. RAS and FLT3 (FMS-like tyrosine kinase 3) mutations, in particular, as well as IKZF1 mutation, can be identified as drivers in B-ALL samples with higher frequency.

## 5. The Regulation of SHIP1

**Figure 4:**
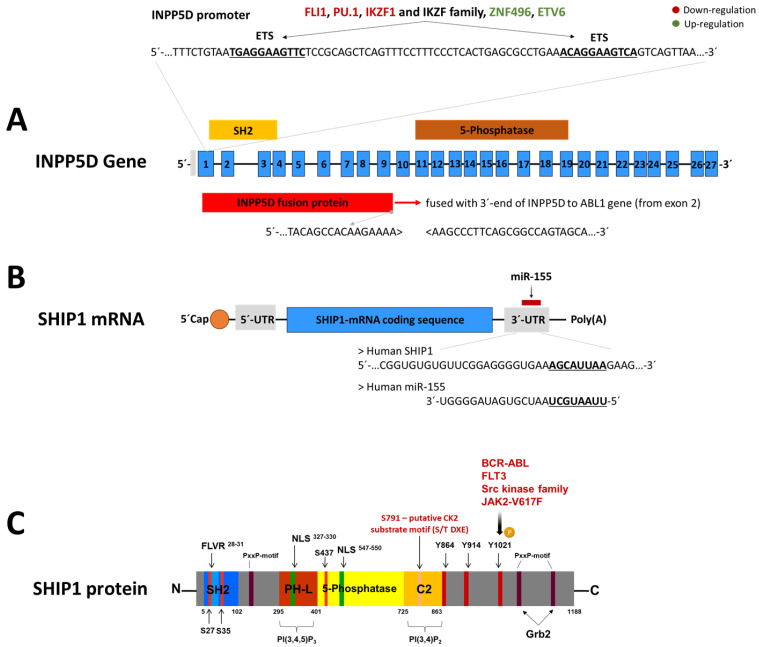
**Illustration of the INPP5D gene, SHIP1 mRNA, and SHIP1 protein structure with various regulation factors.** (**A**) Representation of the full-length INPP5D gene (exon 1–27 in blue). The representation is based on human INPP5D from ENSEMBL. The encoded domains are colored yellow for the Src-Homology 2 (SH2) domain and orange for the 5-phosphatase domain. Above, the promoter region of INPP5D lies in exon 1. The INPP5D promoter contains two highlighted ETS binding sites for putative transcription factors such as FLI1 (friend leukemia integration 1 transcription factor), PU.1 (Spi-1 proto-oncogene), IKZF1, IKZF family members, ZNF496, or ETV6 (ETS Variant Transcription Factor 6). Below, for SHIP1 fusion protein (red), exons 1–9 of INPP5D are fused to the 3′-part of the fusion partner (e.g., with ABL1). (**B**) Micro RNA-155 represses SHIP1 expression through 3′-UTR interactions. (**C**) Representation of the full-length SHIP1 protein with its binding and phosphorylation partners, according to [11,40,91,96,97,98].

The regulation of SHIP1 expression is an important mechanism in leukemic cells for altering the cellular phenotype. It appears to be a more common event in leukemia than mutation of the INPP5D gene. In the following, the known regulatory factors and mechanisms that influence the INPP5D gene, the SHIP1 mRNA, or the SHIP1 protein are summarized (Figure 4).

### 5.1. Regulation of SHIP1 Protein by Tyrosine and Serine Kinases

SHIP1 was barely detectable in BCR-ABL1 (breakpoint cluster region/abelson murine leukemia viral oncogene homolog 1)-positive leukemia cells from patients with chronic myeloid leukemia, since the expression of SHIP1 was suppressed by BCR-ABL1 [99,100]. Moreover, it was shown that the doxycycline-dependent induction of BCR-ABL1 expression correlated directly with the reduced SHIP1 expression in a Ba/F3 cell system [99]. The SHIP1 half-life was reduced from more than 18 h to less than 3 h by inducing BCR-ABL1 expression [99]. The BCR-ABL1 mediated degradation of SHIP1 was specific, as the expression of PTEN or SHIP2 was not affected [74]. In addition, recent studies have shown that SHIP1 is negatively regulated by BCR-ABL1 in both chronic myeloid leukemia (CML) and Philadelphia (Ph)-chromosome-positive B-ALL cells. Inhibitory experiments with the tyrosine kinase inhibitor imatinib led to an upregulation of SHIP1 at the mRNA and protein level in both BCR-ABL1-positive CML and B-ALL cells [11]. In particular, the Src kinases LYN, HCK, and FGR were activated after BCR-ABL1 induction in B-ALL cells [101]. It was also shown that members of the Src kinase family can regulate SHIP1 by phosphorylation and thus have an influence on the PI3K/AKT signaling pathway [41]. However, SRC-mediated tyrosine phosphorylation of SHIP1 is not required for recruitment to the membrane, but only occurs after recruitment to the membrane [102]. Due to the constitutive activation of the FLT3 receptor, the Src kinase family is also activated [103,104,105].

Within the various subtypes of ALL, patients with a BCR-ABL1-positive background, as well as with a genetic change in the KMT2A gene, have by far the worst survival probabilities [106]. Interestingly, both subtypes are characterized by highly activated tyrosine kinases, namely BCR-ABL1 (Ph-positive) and FLT3 (KMT2A-r). It was recently shown that constitutively activated Src kinases downstream of BCR-ABL1 and FLT3 reduce the SHIP1 expression in a SHIP1-Y1021 phosphorylated-dependent manner, with subsequent ubiquitin marked proteasomal degradation [41]. In this context, inhibition of BCR-ABL1 (imatinib), FLT3 (quizartinib), or the Src kinase family (saracatinib) leads to significant reconstitution of SHIP1 protein expression.

In addition to the members of the Src kinase family and BCR-ABL1, SHIP1 was also shown to be phosphorylated by JAK2 [40,107]. Myeloproliferative neoplasms (MPN) often carry the gain-of-function mutation V617F of the Janus kinase 2 (JAK2), which leads to a constitutive activation of the downstream signaling cascade. JAK2 was also identified as a negative regulator of SHIP1 expression in MPN cells, and inhibition of JAK2-V617F implicates a reconstituted SHIP1 expression [40]. This seems to be relevant, because MPN-patient samples frequently showed a loss or a reduction in SHIP1 protein expression. In consequence, the reconstitution of SHIP1 expression in MPN cells leads to decreased cell growth.

Casein kinase 2 (CK2) is a key regulator of cell proliferation and survival in almost all hematological cancers and is often constitutively activated in tumors and acute leukemias [108]. High CK2 protein concentrations are associated with pathological CK2 functions in cancer [108]. The CK2 inhibitor CX4945 has already been tested clinically and led to a strong reduction in SHIP1 expression [11,109]. In addition, a 145 kDa protein may be identified as a potential substrate of CK2. According to the CK2 substrate motif, which obeys the S/T DXE sequence motif [110,111], the putative phosphorylation site of SHIP1 could be located at serine residue 791 (S^791^DPEY) [11]. The inositol phosphate multi-kinase (IPMK), which is associated with the mTOR-Raptor complex, was also previously identified as a substrate of CK2 [112] and a CK2-mediated reduction in Src kinase family activity was shown [113].

### 5.2. Regulation of SHIP1 mRNA by miR-155

KMT2A (Histone-lysine N-methyltransferase 2A)-rearrangements are usually associated with high expression levels of the FLT3 receptor [114,115,116]. The increased expression of the FLT3 receptor frequently leads to constitutive autophosphorylation [117]. In both FLT3-ITD-positive AML and KMT2A-AFF1-positive B-ALL, the constitutive activation of the FLT3 receptor leads to a strong overexpression of the microRNA miR-155. Repression of SHIP1 can be caused by oncogenic microRNAs such as miR-155, which binds directly to the 3′-UTR of SHIP1 mRNA and inhibits translation [96,118,119]. MicroRNAs are short (about 20 nucleotides), single-stranded RNA molecules that regulate gene expression. Upregulation of miR-155 and repression of SHIP1 were first observed in patients with B-cell lymphoma, and low SHIP1 expression was correlated with poor patient survival [120].

Studies in transgenic mice in which miR-155 expression is induced at the late stage of pro-B cell development initially show pre-leukemic proliferation of pre-B cells and later develop lymphoblastic leukemia/lymphoma [121]. In these transgenic mice, the highest expression of miR-155 is the origin of the leukemia and is directly linked to the gradual downregulation of SHIP1 [122,123]. Src kinases directly regulate SHIP1-mediated degradation at the protein level, as described before, or can increase miR-155 transcription via NF-κB (nuclear factor kappa-light-chain-enhancer of activated B-cells), STAT5 (signal transducer and activator of transcription 5), or interferon regulatory factor 4 (IRF4), thereby reducing SHIP1 mRNA expression [96,124,125,126].

### 5.3. Transcriptional Regulation of INPP5D

The transcription factors from the Ikaros family, Helios and Ikaros, also influence the expression of SHIP1 [97]. Dominant-negative isoforms of the transcription factor Ikaros (IKZF1) were found at a high degree in Ph-positive B-ALL cases [127,128,129]. Ikaros can bind to the promoter of the INPP5D gene and has a repressive effect on SHIP1 expression in DT40 B cells, whereas Helios has an activating function [97]. In contrast, Song and coworkers showed that Ikaros represses the transcription of genes of the PI3K signaling pathway in Nalm-6 B-ALL cells, but promotes the transcription of SHIP1 [130]. Molecular and pharmacological inhibition of casein kinase 2 (CK2) increases the activity of Ikaros as a transcriptional regulator [130]. Inhibition of the antagonistic CK2 and targeted restoration of Ikaros function led to a strong reduction in SHIP1 expression and at the same time to a significant inhibition of AKT activation and cell growth [11]. Importantly, the tumor suppressive function of Ikaros was enhanced by a SHIP1-dependent additive effect. In a recent model of the transcriptional regulation of SHIP1 by Ikaros in B-ALL cells, SHIP1 possessed two Ets binding sites for binding Ikaros and Ikaros family members. Ikaros homodimers led to the repression of the INPP5D expression, while Ikaros dominant-negative isoforms were no longer able to maintain the inhibition of transcription [11]. Furthermore, Ikaros family members or other C2H2 zinc finger are proposed to lead to different effects on transcription and modulate Ikaros-dependent transcription. Interestingly, ZNF496 is among the significantly enriched transcription factors associated with INPP5D gene expression [11]. ZNF496, like IKZF1, is a C2H2 zinc finger [131]. It is possible that IKZF1 (or other IKZF family members) and ZNF496 could also cooperate to regulate the gene expression of INPP5D.

Recent data showed that SHIP1 expression was upregulated at RNA and protein level in a B-ALL subgroup with ETV6-RUNX1 (ETS variant transcription factor 6/runt-related transcription factor 1) translocation. ETV6 belongs to the Ets family of transcription factors [132,133]. Direct regulation of SHIP1 mRNA expression by ETV6 has not yet been demonstrated. In a recent model, fusion with RUNX1 prevented the ETV6 transcription factor from binding to the Ets binding site in the INPP5D locus, thus abolishing the ETV6-mediated repression of SHIP1 expression [11]. This hypothesis was supported by the finding that FLI1, another member of the Ets transcription factor family, was shown to bind directly to the Ets binding site within the INPP5D promoter, thus directly blocking SHIP1 transcription [98,134]. The ETS family transcription factor PU.1 also inhibits the transcription of SHIP1 [135].

## 6. Expression Status of SHIP1 in the Different Subtypes of ALL

Acute lymphoblastic leukemia (ALL) is the most common cancer and the most common cause of cancer-related death in childhood. Despite significantly improved chances of cure, the prognosis for patients from a high-risk group and patients with relapse remains poor. The heterogeneity of childhood ALL make a general therapeutic strategy more difficult and has led to subtype-specific treatment approaches [136]. Up to now over 20 subtypes have been described based on cytogenetic analysis, immunophenotyping, and gene expression profiles [137].

Chromosomal translocations are among the most important genetic aberrations that are causally involved in the development of B-ALL. More than 30 different, non-random translocations are known for ALL. These translocations can result in chimeric fusion proteins that often include a kinase or a transcription factor [137]. As a result, these fusion proteins lead to aberrant signal transduction (activated kinase) or dysregulated transcription, which, in part, lead to very different gene expression profiles. It was shown that the inositol-5-phosphatase SHIP1 is differentially expressed across all ALL subtypes [138].

Different subtypes known so far for B-ALL include DUX4-r, ETV6-RUNX1, ETV6-RUNX1-like, hyperdiploid, KMT2A-r, low hyperdiploid/near triploid, near-haploid, MEF2D-r, PAX5-alt, PAX5-P80R, Ph-like, Ph-positive, TCF3-HLF, TCF3-PBX1, ZNF384-r, and iAMP21 [137]. In the next section, the most common and important subgroups of ALL are briefly summarized and the expression status of SHIP is described (Table 2).

### 6.1. ETV6-RUNX1 Subroup

The t(12;21)(p13;q22) ETV6-RUNX1 translocation (formerly known as TEL-AML1) predominantly occurs in pediatric cases and comprises approximately 20–25% of children and up to 3% of adults with B-ALL [139]. Notably, cells with ETV6-RUNX1 translocation can also be detected by PCR in 5% of healthy newborns, but do not transform into overt leukemia [140]. This translocation is of favorable outcome, but up to 20% of patients with ETV6-RUNX1 were shown to relapse [141,142]. RAS and WHSC1 mutations are a key feature of this subgroup [137].

ETV6 is a transcription factor and a member of the ETS transcription factor family. It regulates the development and growth of diverse cell types, particularly those of hematological tissues. RUNX1 is a nuclear transcription factor that regulates the differentiation of hematopoietic stem cells into mature blood cells [143]. It belongs to the Runt-related transcription factor (RUNX1) family of genes, which are also called core binding factor-α (CBFα). The runt domain, which is important for DNA binding, shows homology to the p53 family. RUNX1 proteins form a heterodimeric complex with CBFβ, which confers increased DNA binding and stability to the complex. Disruption of either RUNX1 or CBFβ in mice leads to embryonic lethality [144], since RUNX1 is essential for the development of HSCs in the embryo [145,146].

Blood taken at birth demonstrates that ETV6-RUNX1-driven leukemia is initiated in utero, but does not transform into overt leukemia until the third or fourth year of life, with the notion that additional molecular hits are required for malignant transformation [147,148]. In addition, altered cytokine environments associated with abnormal immune responses due to strong infection may increasingly eliminate normal pre-B cell clones from the repertoire and favor the selective outgrowth of pre-B cell clones that already have a pre-leukemic genetic lesion. ETV6-RUNX1-inducible B precursor cells show slower proliferation than their non-induced counterparts, but are also more resistant to inhibition of proliferation by TGFβ [149].

INPP5D gene expression and SHIP1 protein expression was highly upregulated in a pediatric ETV6-RUNX1 subgroup [11]. In agreement, the dataset of Kohlmann et al. showed markedly increased SHIP1 expression in ETV6-RUNX1-positive B-ALL compared to healthy hematopoietic cells [150]. The INPP5D expression in the Haferlach dataset [151] also shows increased expression in the ETV6-RUNX1-positive B-ALL group compared to the ETV6-RUNX1-negative B-ALL group. Accordingly, SHIP1 expression could be promoted by the expression of the ETV6-RUNX1 fusion protein.

### 6.2. TCF3-PBX1 Subgroup

Transcription factor 3 (TCF3) is involved in two types of translocations, t(1;19)(q23;p13) and t(17;19)(q22;p13), which result in TCF3-PBX1 and TCF3-HLF. The TCF3-PBX1 translocation (formerly known as E2A-PBX1) is found in approximately 3-5% of both children and adults with B-ALL [139]. This translocation has an intermediate outcome with intensive therapy [152]. It is also associated with central nervous system (CNS) relapse [153]. Mutations of the TP53 gene and in components of cytokine signal transduction, such as the IL7 receptor, are key features of this subgroup [154]. TCF3-HLF is a rare gene fusion (<1% of ALL), and relapse and death events are common [155,156].

Transcription factor 3 is a member of the E protein family of helix-loop-helix transcription factors. E proteins activate transcription by binding to regulatory E-box sequences on target genes as heterodimers or homodimers. This gene regulates many developmental patterning processes, such as lymphocyte and CNS development. Heterodimers between TCF3 and tissue-specific basic helix-loop-helix (bHLH) proteins play major roles in determining tissue-specific cell fate during embryogenesis [157,158]. The transactivation domains of E proteins and KMT2A proteins are very similar and both bind to CBP [159]. Pre-B-cell leukemia transcription factor 1 (PBX1) is a nuclear protein that belongs to the PBX homeobox family of transcriptional factors with relevance in embryonic development [160]. The resulting fusion protein, in which the DNA binding domain of TCF3 is replaced by the binding domain of PBX1, transforms cells by constitutively activating transcription of genes regulated by the PBX protein family. The gene expression of INPP5D was low in the TCF3-PBX1 subgroup [11].

### 6.3. KMT2A-r Subgroup

Patients with KMT2A-rearrangement (formerly known as MLL) show a clear peak in infancy [139] and rearrangements are found in 70% of infants, 5% of children, and 10–15% of adults.

KMT2A gene rearrangements confer a very poor prognosis in infants and adults, and an intermediate prognosis in children [106]. There are at least 80 known KMT2A fusion partners, but most fusions involve t(11;19)(q23;p13.3) MLLT1, t(9;11)(p21;q23) MLLT3, and t(4;11)(q21;q23) AFF1 in B-ALL. KMT2A-r is frequently associated with a mutation in the FLT3 (fms-like tyrosine kinase 3), NRAS, or KRAS gene [117,161]. In addition, FLT3 is frequently upregulated in cells with KMT2A-r [114,115,116]. Furthermore, this subtype shows a deregulated gene expression of the HOXA gene family and disturbed epigenetics by DOT1L (disruptor of telomeric silencing 1-like) [162,163]. In addition, patients with an KMT2A rearrangement are prone to relapse, in which a clonal selection advantage and an associated enormous expansion of parallel malignant myeloid subclones occur during the treatment of B-ALL cells (lineage switch) [164]. In accordance, KMT2A fusions not only occur in B-ALL, but are also found in AML. The KMT2A gene is critical for the development and maintenance of hematopoietic stem cells (HSCs) and plays a role in embryonic development, hematopoiesis, and neurodevelopment [165].

Overall INPP5D gene expression is not conspicuous, but interestingly is significantly more highly expressed in the pediatric cohort of KMT2A than in the adult cohort of this subgroup [11]. SHIP1 protein was shown to be strongly downregulated in B-ALL cells with KMT2A-rearrangement by high FLT3 activity [41,138].

### 6.4. Ph-Positive and Ph-like Subgroup

The Philadelphia chromosome t(9; 22)(q34;q11) is characteristic for patients with CML (95%). The number of cases of B-ALL with a Ph-positive or Ph-like background increases with age. About 3% of children and 25-30% of adults suffer from a Ph-positive ALL [139]. Ph-positive T-ALL is very rare and only a few cases have been described in the literature [166,167,168].

The Philadelphia chromosome encodes for the BCR-ABL1 fusion protein. ABL1 normally codes for a tyrosine kinase that is located in the cytoplasm. The name BCR gene (breakpoint cluster region) originates from the frequent breaks that occur in this gene. It is a kinase that has not yet been well characterized. Various possible breakpoints can be identified in the BCR gene, which are referred to as m-BCR (minor), M-BCR (major), and µ-BCR (micro) [169]. In contrast, the breakpoint of the ABL1 gene is always in the same intron in chromosome 9. As a result, fusion proteins of different sizes can be formed. These fusion proteins have a size of 190 kDa, 210 kDa, and 230 kDa, respectively. In vitro studies show that p190BCR-ABL1 is a more active tyrosine kinase than p210BCR-ABL1 and patients tend to have a longer duration of remission [170,171,172]. In childhood ALL, the 190 kDa fusion protein is expressed in 90% of cases and the 210 kDa protein in 10% of cases [173]. In almost all cases of CML and in about half of adult Ph-positive ALL, a 210 kDa fusion protein can be identified [174]. In contrast to the ABL1 protein, the BCR-ABL1 fusion protein is constitutively active. The N-terminal region of ABL1, which normally blocks the catalytic domain of the tyrosine kinase, is missing and the BCR coiled-coil domain leads to dimerization and trans-autophosphorylation of the tyrosine kinase [175]. B-lymphoid and myeloid leukemias are often driven by the same oncogenes, such as BCR-ABL1, but differ significantly in their clinical features [176,177]. While more than 95% of patients with chronic myeloid leukemia achieve long-term disease-free survival after treatment with tyrosine kinase inhibitors [176], patients with Ph-positive B-ALL have a significantly worse prognosis and frequently relapse within a short period of time after initial remission or develop resistance [178,179,180,181].

Current and previously developed tyrosine kinase inhibitors strongly select for resistant BCR-ABL1 mutants [182]. IKZF1 mutations are identified in an enriched manner in relapsed patients [183,184]. This includes both the de novo acquisition of an IKZF1 mutation and the selection of a subclone with a severe mutation that was present at a low percentage before the relapse [185,186]. In approximately 83% of Ph-positive ALL cases, but not in CML, Ikaros carries a point mutation (10%) or shows a deletion (90%) [127].

This finding was supported by strong differences in the expression of SHIP1 between Ph-positive B-ALL and CML [187]. Patients with CML showed low SHIP1 protein expression [99], whereas SHIP1 protein was highly upregulated in pediatric BCR-ABL1-positive B-ALL [41]. The differences could not be explained by the expression of BCR-ABL1 alone, since inhibitor treatment with imatinib led to upregulation of SHIP1 in both cell types [41]. Further data [150] show higher INPP5D gene expression in the BCR-ABL1-positive B-ALL subgroup compared to healthy hematopoietic cells.

A total of 10–15% of children and up to 25% of adults suffer from a Ph-like ALL [186]. The gene expression profile of Ph-like ALL leukemia cells is similar to that of Ph-positive ALL cells [188]. However, the patients do not carry the BCR-ABL1 translocation. The genetic changes, on the other hand, are very diverse and also lead to the activation of tyrosine kinase signaling. More than half of the cases of Ph-like ALL show an overexpression of CRLF2, whereby almost half of the cases show a JAK-STAT mutation (often JAK2-R683G) and a concomitant activation of the JAK-STAT signaling pathway [189]. JAK mutations are significantly associated with alterations of the Ikaros gene and deletions of CDKN2A/B (cyclin dependent kinase inhibitor 2A/B) [190,191]. In the Ph-like ALL group without CRLF2 (cytokine receptor like factor 1) overexpression, fusion proteins with JAK, ABL1, and other tyrosine kinases are frequently found [189].

The INPP5D gene expression is significantly higher in the pediatric cohort of Ph-positive and Ph-like subgroups than in the adult cohorts of these subgroups [11]. This suggests an age-specific expression of SHIP1.

### 6.5. T-ALL

So far, ten subtypes of T-ALL have been delimited according to their molecular features: G1 (LYL1/LMO2 overexpression), G2 (GATA-3 mutation), G3 (SPI1-fusion), G4 (KMT2A-rearrangement), G5 (MLLT10-rearrangement), G6 (HOXA10-fusion), G7 (TLX3-overexpression), G8 (TLX1-overexpression), G9 (NKX2-1-overexpression), and G10 (TAL1/LMO1-overexpression) [192]. The SHIP1 gene INPP5D is strongly expressed in G1 and forms a cluster with the genes of the G1 subgroup (LYL1/LMO2), as well as with NOTCH1 (neurogenic locus notch homolog protein 1), ETV6, and MEF2C (myocyte-specific enhancer factor 2C) [138]. In contrast, INPP5D is significantly downregulated in the G10 subgroup. The activity profile of the tyrosine kinase receptors NTRK (neurotrophic receptor tyrosine kinase 1) and PDGFR (platelet-derived growth factor receptor), which are upregulated in T-ALL subgroups with low SHIP1 expression, are significantly disabled after SHIP1 reconstitution. Interestingly, PDGFRB (G2, G6 and G7), IGF1R (insulin-like growth factor 1 receptor) (G2 and G9), and FGFR1 (fibroblast growth factor receptor 1) (G5–G10) showed higher expression in subgroups with low INPP5D expression (G1) [138]. This strongly suggests that inhibition of the corresponding RTKs in the specific T-ALL subgroups with low INPP5D expression is required to compensate for the loss of SHIP1 expression.

Moreover, the inositol phosphatase PTEN, which is an important tumor suppressor, is also downregulated in one third of the T-ALL cell lines and in two thirds of primary T-ALL samples [193]. In connection with the frequent functional loss of PTEN due to mutations [194] or via post-transcriptional and post-translational mechanisms [9,195,196,197], increased AKT activity can be observed in T-ALL [10].

Lo et al. described that the T-ALL cell line Jurkat does not express SHIP1 protein, because the two SHIP1 alleles are mutated. One allele has a nonsense mutation in codon 345 and the second allele shows a deletion of the first 47 bp of exon 12, resulting in a frameshift and premature stop [193]. Primary T ALL cells also frequently harbor SHIP1-inactivating mutations. In contrast, the COSMIC database only contains an entry for a silent mutation K420K for SHIP1 in the Jurkat cell line [53]. Transcriptomic studies using microarray analyses have also confirmed that no SHIP1 mRNA can be detected in 65 of 79 T-ALL patient cells examined (82%) (absent call) [138]. In contrast to other reports, it was shown for the first time that SHIP1 is not lost in T-ALL cells, but is strongly downregulated [138]. SHIP1-mRNA expression is frequently reduced in primary T-ALL samples, which is recapitulated by the decrease in SHIP1 expression at the protein level in seven out of eight T-ALL patient samples. Reduced expression of SHIP1 leads to an increased activation of the PI3K/AKT signaling pathway. Reconstitution of SHIP1 expression in Jurkat cells points to a decreased cellular proliferation after transplantation into NSG mice [138]. Together, these findings show that full-length SHIP1 is expressed in T-ALL samples.

Accordingly, SHIP1 could be detected as weakly expressed at the protein level at 145 kDa in the Jurkat cell line [138]. The Jurkat cells used in this study must therefore have had at least one SHIP1 allele without a nonsense mutation, since endogenous SHIP1 expression of the full-length protein was detected here. The weak expression of SHIP1 in Jurkat cells is comparable to the expression of SHIP1 in the CML cell line K562. Rather, the weak SHIP1 expression can be attributed to the presence of the BCR-ABL1 fusion protein in K562 cells [99]. Interestingly, a BCR-ABL1 fusion transcripts in the T-ALL cell line Jurkat was reported using a two-step RT-PCR procedure [198]. Notably, reconstitution of SHIP1 expression by lentiviral transduction in the Jurkat cell line shows that SHIP1 is degraded quickly at the protein level, but is stable at the mRNA level [138]. The frequency of T-ALL cells with weakly expressed SHIP1 protein is very high. This could therefore probably be a general cause of this effect. A number of deregulated genes with activating mutations or translocations for T-ALL have been described: FBXW7 (14%), TAL1 (30%), NOTCH1 (50%), CDKN2A (61%), and CDKN2B (58%) [194]. In addition, an association of NOTCH1 mutations with FLI1 overexpression has been described [199]. In addition, the constitutive activation of the T-ALL oncogenes NOTCH1 and TAL1 by mutation or translocation often leads to a strong misregulation of the expression of various microRNAs (miR-19b, miR 20a, miR-26a, miR-92, and miR-223), which jointly downregulate the expression of Ikaros, PTEN, and FBXW7 [200,201]. In addition, NOTCH1 activates the NF-KB signaling pathway in T-ALL [202,203], and NF-κB can subsequently increase the transcription of miR-155 and thus repress the expression of SHIP1 mRNA [124].

Jurkat cells harbor a NOTCH1-ITD mutation, which results in high expression levels of the ICN1 protein and activation of the Src kinase family [204]. Compared to the phosphorylation of the Src kinase family in the K562 cell line, the phosphorylation in the Jurkat cell line is significantly stronger, despite the presence of BCR-ABL1 and the associated activation of the Src kinase family in the K562 cell line [41]. Inhibition of the Src kinase family leads to an increase in SHIP1 expression [41]. This suggests, at least in part, an active degradation of the SHIP1 protein by tyrosine kinases in the Jurkat cell line. Thus, there could also be several additive effects affecting SHIP1 expression in T-ALL.

## 7. Beyond Leukemia: SHIP1 Expression in Carcinoma

SHIP1 expression can be identified in 49 out of 72 tumor entities in a multi-tumor tissue microarray [205]. SHIP1 appears to be primarily expressed in diseased intestinal tissue. In detail, SHIP1 expression can be identified in 62% of cases with colorectal carcinoma [205]. Moreover, the protein expression of SHIP1 was identified in 6 out of 12 carcinoma cell lines examined (Sk-ChA1, EGI-1, HepG2, HT-29, SW-480, and WM1366 cells) [206]. Lung cancer cells expressing SHIP1 show a growth disadvantage compared to cells without SHIP1 expression [56,207]. In addition, overexpression of SHIP1 in lung cancer cells in vivo leads to reduced growth, migration, and invasion [207]. Clinical data show that lung cancer patients with reduced SHIP1 expression have a reduced survival rate, in contrast to patients with high SHIP1 protein levels [207]. The expression of SHIP1 in lung carcinoma cells can be attributed to the absence of CpG islands in the promoter region of the gene [207]. In addition, colon cancer patients with high SHIP1 mRNA expression have a lower risk of relapse than patients with low SHIP1 expression [208].

It was also shown that SHIP1 is more strongly expressed in metastatic colon carcinoma cells than in the primary cancer cells, which allows for an increase in AKT signaling in metastatic cells, giving them an advantage from an evolutionary point of view [206]. Therefore, SHIP proteins are more than just tumor suppressors or oncogenes [76]. Rather, they involve finely balanced protein expression to adjust oncogenic signaling in malignant cells. Mechanistically, the increased SHIP1 expression reduces the activation of the PI3K/AKT signaling to a value that is below the threshold that leads to cell death. This mechanism gives the cell a selection advantage. The results showed that genetic hyperactivation of PI3K/AKT-signaling or blocking the activity of the inhibitory phosphatase SHIP1 induces acute cell death in CRC cells, because of excessive accumulation of reactive oxygen species [206]. This study demonstrated that CRC cells critically depend on mechanisms to fine-tune PI3K/AKT activity. In accordance, acute hyperactivation of the B cell kinase SYK in melanoma cells can induce cellular senescence [209]. Loss of INPP4B and PTEN in dermal endothelial cells also leads to cellular senescence [210]. In addition, hyperactivation of AKT, as a result of pharmacological inhibition of PTEN in a human xenotransplantation model for prostate cancer, led to inhibition of tumorigenesis [211]. Acute hyperactivation of kinase signaling in combination with excessive proliferation, cellular stress, and activation of a DNA damage response can trigger oncogene-induced senescence, a mechanism that prevents malignant transformation in cells [212,213,214,215]. Therefore, recent results have shown that titration of RAS altered senescence state and influenced tumorigenesis [216]. However, the thresholds for induction of cell death are probably much higher in non-hematopoietic cell entities than in B cells.

## 8. Ikaros Regulates SHIP1 Expression and Influences Cell Metabolism by Mediating the AKT Pathway

The modern role of SHIP1 and its fine-tuning of expression is evident in current research in ALL in the context of B cell activation or its oncogenic mimicry. B cells are highly dependent on the survival and proliferation signals emanating from their antigen receptor (B cell receptor; BCR). Of the BCR-dependent signaling cascades, the PI3K/AKT signaling pathway plays a prominent and central role. Thus, BCR-deficient B cells in combination with the specific activation of PI3K/AKT signaling downstream of the BCR show that cell survival can only be rescued by this one signaling pathway [217]. Moreover, the PI3K/AKT signaling pathway was shown to play a critical role in B-ALL pathogenesis, progression, and resistance development by driving lipid metabolism, ferroptosis, glutathione metabolism, and coagulation cascade [218].

B cells are subject to an active negative selection process [219]. Pre-B cells are under strong selective pressure and frequently suffer DNA damage during the various rounds of recombination [220]. As a result, up to 75% of early human B cell precursors express self-reactive B cell receptors [221]. Both weakening of the BCR signal strength below a minimum threshold (non-functional BCR) and hyperactivation above a maximum threshold (autoreactive BCR) can lead to cell death and elimination of B-cell clones in the early stages of B-cell development [187].

**Figure 5:**
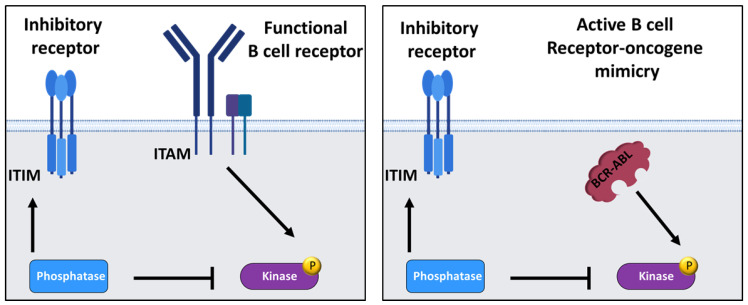
**The influence of kinases and phosphatases on the signaling strength of the B cell receptor or its oncogenic mimicry.** Inhibitory phosphatases downstream of a functional B cell receptor or its oncogenic tyrosine kinase mimicry of a non-functional receptor can attenuate the signaling strength upon recruitment to ITIM-bearing inhibitory receptors. In consequence, certain phosphatases act as antagonists of constitutively activated kinases and thus BCR signaling. Increased expression of inhibitory phosphatases could raise the threshold for autoimmunity checkpoint activation and help autoreactive B cells evade negative selection, according to [187].

While pre-B-ALL clones often do not express a functional pre-BCR, oncogenic tyrosine kinases (e.g., BCR-ABL1) and mutations of components of the RAS or PI3K/AKT pathway can also mimic the survival and proliferation signals of a constitutively active pre-BCR (Figure 5) [1,2]. These observations led to the concept that targeted hyperactivation of BCR signaling above a maximum threshold by hyperactivation of one or more downstream components of the B cell receptor signaling pathway represents the functional equivalent of autoimmunity checkpoint (AIC) activation of a self-reactive BCR, and thus induces negative selection of transformed cells [95,187,222]. Phosphatases often serve as antagonists of BCR signaling and the signaling of activated kinases (such as SYK, PI3K, and ERK) after recruitment to ITIM-bearing inhibitory receptors (such as LAIR1, PECAM1, and CD300A) [12]. Recent studies have shown that particularly high expression levels of inhibitory phosphatases (such as SHIP1, PTEN, and DUSP6) in leukemia allow cells with strong oncogenic signaling to escape negative selection by attenuating signal strength [95,187,222,223]. For this reason, intensive research is currently being carried out into new specific SHIP1 inhibitors [224,225,226,227,228].

Moreover, pre-B-ALL clones often carry genetic lesions of the transcription factors PAX5 (paired box protein 5), IKZF1, and EBF (early B cell factor) [127,229]. These transcription factors control the expression of pre-BCR components [230], but they are also responsible for the transcriptional repression of glucose and energy metabolism [230]. Normal B cells frequently acquire or develop potentially oncogenic lesions in early childhood and later in life. For example, pre-leukemic B cell clones with oncogenic lesions (BCR-ABL1, KMT2A-AFF1, and ETV6-RUNX1) are frequently found in cord blood samples from healthy newborns [231,232,233,234]. In addition, silent oncogenes such as BCR-ABL1 and KRAS are frequently found in small fractions of normal B cells in healthy adults and children [198,235,236]. The crucial gatekeeper function of IKZF1 was further illustrated in two cases of identical twins with prenatal-acquired BCR-ABL1 fusion protein in a portion of their newborn B cells, where one of both twins in each case additionally acquired a postnatal IKZF1 deletion, which led to transformation into overt and fatal B-ALL (previously free of leukemia cells like the other twin) or to death after B-ALL disease (the other twin went into remission after treatment) of the respective twin [233].

In accordance with this, gene expression analysis revealed that SHIP1 exhibited a substantial influence, through regulating AKT, on the energy metabolism and cellular stress response in B-ALL cells [11]. Oncogenic kinases highly consumed ATP and increased the energy requirement of pre-malignant B cells. In accordance with this, malignant cells have increased glucose uptake and accelerated glucose metabolism [230]. Thus, ATP restriction served as a safeguard against the elimination of these cells. IKZF1 was shown to regulate genes associated with glucose transport and to limit glucose and energy supply in B cells to levels insufficient for malignant transformation [230,234,237,238]. Accordingly, the glucose transporters GLUT1, GLUT3, GLUT6 (glucose transporter 1/3/6), and hexokinase 2 (HK2), and the insulin receptor INSR are transcriptionally suppressed, and factors that negatively regulate glucose transport (NR3C1, CNR2, HK2, and TXNIP) are transcriptionally activated [230]. Thus, IKZF1 sets the threshold for responses to glucocorticoids [230,239] and dominant-negative mutants of IKZF1 and PAX5 alleviate glucose and energy restriction [230]. The PI3K/AKT signaling pathway has a high energy requirement [240] and phosphorylation of NR3C1 (nuclear receptor subfamily 3 group C member 1) by AKT inhibits the nuclear translocation of the glucocorticoid receptor [241]. Glucocorticoids act via the nuclear hormone receptor NR3C1, pharmacologically suppress glucose uptake, inhibit glucose transporters such as GLUT1, and induce energy stress in B cells [230]. Furthermore, the PI3K/AKT pathway regulates the expression and mitochondrial localization of HK2, which phosphorylates glucose to generate glucose-6-phosphate [242].

**Figure 6:**
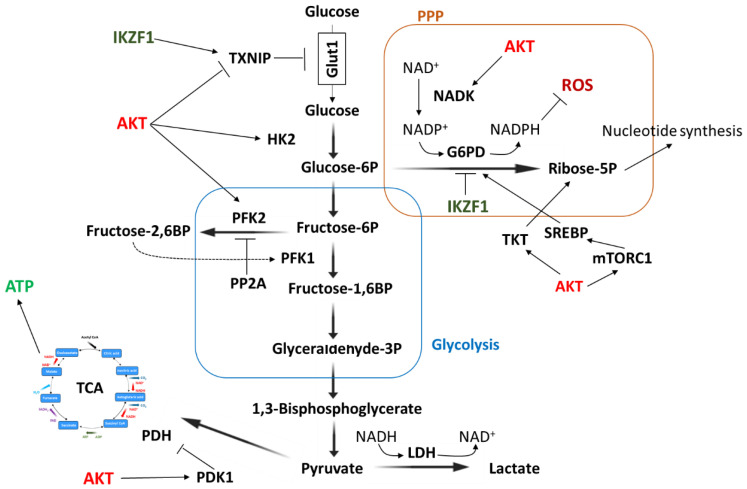
**Model of the contrary regulation of the energy metabolism by Ikaros and the PI3K/AKT/mTOR signaling pathway.** AKT has a high energy requirement and stimulates key metabolic enzymes by directly phosphorylating them. AKT promotes membrane localization of glucose transporter 1 (GLUT1) and inhibits endocytosis of GLUT1 by phosphorylation of the thioredoxin-interacting protein (TXNIP), which increases glucose uptake. Furthermore, AKT activates hexokinase 2 (HK2), which phosphorylates glucose to generate glucose-6-phosphate (glucose-6P). Glucose-6P cannot be transported out of the cell and servers as metabolite for the pentose phosphate pathway (PPP) and glycolysis. The generation of NADPH for antioxidant protection occurs through the G6P dehydrogenase (G6PD) enzyme, which becomes activated by the AKT/mTORC1/SREBP axis. The AKT-dependent activation of nicotinamide adenine dinucleotide (NAD) kinase (NADK) leads to the catalyzation of NAD+ to NADP+, a precursor of NADPH. AKT also activates the non-oxidative PPP enzyme transketolase (TKT), which leads to ribose-5-phosphate production for nucleotides synthesis. Inhibition of G6PD can lead to massive cell stress by increasing reactive oxygen species (ROS) levels. AKT phosphorylates and activates Phosphofructokinase-2 (PFK-2), a domain of the bifunctional enzyme 6-phosphofructo-2-kinase/fructose-2,6-biphosphatase (PFKFB2). Fructose-6P becomes converted to fructose-2,6-bisphosphate (fructose-2,6BP), which represents an allosteric activator of phosphofructokinase 1 (PFK1). Increased PFK1 conversation of fructose-6P to fructose-1, 6BP favors the flux of glucose carbon through glycolysis at the expense of the PPP. PP2A is essential for maintaining the balance between glycolysis and PPP by dephosphorylation and reducing activity of PFK2. AKT phosphorylates PDK1, which inhibits the pyruvate dehydrogenase (PDH). PDH initiates the oxidation in the mitochondria using the tricarboxylic acid (TCA) cycle. In consequence, pyruvate as the end product of glycolysis will be more commonly converted to lactate by the lactate dehydrogenase (LDH). This process also leads to regeneration of NAD+, which in turn is needed for the PPP. In contrast, the glucose transporters GLUT1/3/6, the insulin receptor INSR, and the glucose metabolism effector G6PD are transcriptionally suppressed, whereas the cannabinoid receptor 2 (CNR2), the glucose transport inhibitor TXNIP, and the nuclear hormone receptor NR3C1 are transcriptionally activated by IKZF1. Glucocorticoids act via the nuclear hormone receptor NR3C1, suppress glucose uptake, and inhibit glucose transporters such as GLUT1. Phosphorylation of NR3C1 by AKT inhibits the nuclear translocation of the glucocorticoid receptor.

Ikaros and the PI3K/AKT/mTOR signaling pathway had opposite functions in the regulation of the glucose-metabolism effector G6PD (glucose-6 phosphate dehydrogenase), the glucose transport inhibitor TXNIP (thioredoxin interacting protein), and the energy stress sensor AMPK (AMP-activated protein kinase) (Figure 6) [11]. In addition, phosphatases are also able to stabilize energy reserves and thus prevent negative selection of the cell [95,187,222]. Targeted inhibition of phosphatases leads the cell into energy crisis and ultimately to cell death [95]. Recent studies have shown that the restriction of nutrient supply represents a limitation for the malignant transformation of B cell precursors [230,237,243]. Compared to children with normal glucose and insulin levels, obese children or children with high blood sugar levels show a higher relapse in B-ALL and also have significantly worse outcomes [244,245].

Indeed, SHIP1 had an antagonistic effect on the downstream signaling of the B cell receptor, as demonstrated by the reduced activation of the AKT signaling cascade in B-ALL cells with high SHIP1 expression [11]. Recent studies have shown that Ikaros sets the threshold for negative B-cell selection by regulation of SHIP1, and thereby controls the signaling strength of the AKT pathway [11]. High SHIP1 expression in both Ph-positive and ETV6-RUNX1 B-ALL cells could potentially contribute to B-cell acute lymphoblastic leukemia (B-ALL) by raising the threshold for AIC activation, allowing malignant cells with strong oncogenic B-cell receptor signaling to escape negative selection [11,187]. The restoration of Ikaros wild-type expression reduced strong SHIP1 expression to a moderate level, thereby lowering the threshold to escape negative selection, and led to a significant reduction in cell growth and increased apoptosis [11].

Importantly, the tumor suppressive function of Ikaros was enhanced by a SHIP1-dependent additive effect. Pharmacological inhibition of the antagonistic CK2 led to a strong reduction in SHIP1 expression and at the same time to a significant inhibition of AKT activation and cell growth [11]. In line with this, it was reported that the dominant-negative Ikaros isoform protects ALL cells from apoptosis by manipulating the AKT/FoxO1-axis (forkhead box-protein O1) [246]. In the present model, B-cell-specific negative selection against cells with hyperactive tyrosine kinase signaling could be undermined by the dominant-negative Ikaros isoform and could raise the threshold to be able to induce cell-intrinsic apoptosis [11].

## 9. Discussion

Acute lymphoblastic leukemia (ALL) of childhood can often be traced back to a preleukemic clone with a prenatal genetic lesion [148,232]. However, less than 1% of newborns carrying such a genetic lesion (ETV6-RUNX1 or BCR-ABL1) develop ALL [147]. Postnatally, preleukemic clones can also acquire secondary mutations or show deregulated protein expression and then develop into overt leukemia [247,248,249,250].

Particularly high expression levels of inhibitory receptors or phosphatases are frequently found in B cell receptor-directed ALL [11,95,187,222,223]. The heterogeneity of the ALL subtypes makes a general therapeutic strategy that directly targets the important inhibitory phosphatase SHIP1 difficult, since SHIP1 is differently expressed in the various subgroups of ALL. In ETV6-RUNX1 and Ph-positive B-ALL cells, SHIP1 appears to be strongly upregulated, while SHIP1 appears to be strongly downregulated in B-ALL cells with KMT2A translocation and in T-ALL [11,138]. One reason for the strong SHIP1 expression in a subset of B-ALL cells could be that malignant B cells have a survival advantage in B cell selection, due to a strong feedback loop via BCR signaling. A more detailed analysis shows that reconstitution of Ikaros wild-type expression can reduce the strong expression of SHIP1 to an appropriate level, and thus lowers the threshold for possible negative cell selection. It can also be assumed that the regulation of SHIP1 expression by Ikaros and the Ikaros family members is dynamic. Depending on the level of SHIP1 expression and the B cell receptor signaling strength, Ikaros may detect these and dynamically change gene expression to act as a tumor suppressor [251]. Thus, results demonstrate that ALL cells critically depend on mechanisms to fine-tune SHIP1 expression as an important antagonist of the PI3K/AKT pathway. A possible pharmacological intervention could therefore aim at restoring Ikaros activity in ALL cells with dominant-negative Ikaros isoforms. Rather, it can be shown that the expression and thus also the activity of SHIP1, especially in high-risk groups, is regulated by aberrantly activated tyrosine kinases such as BCR-ABL1 and FLT3, with downstream signaling of the Src kinase family. In summary, recent data point to a regulatory window for the expression level of SHIP1, which must neither be undercut (e.g., in T-ALL and CML) nor exceeded (e.g., in Ph-positive B-ALL and ETV6-RUNX1-positive B-ALL). The regulatory function of Ikaros in SHIP1 expression in B-ALL highlights the relevance of the AKT pathway as a therapeutic target for the treatment of this disease.

## Figures and Tables

**Table 1:** Comparison of the frequency of mutations in the major components of the PI3K/AKT/mTOR, RAS, and BCR signaling pathway in carcinoma and leukemia [taken from https://cancer.sanger.ac.uk/cosmic (accessed on 7 June 2025)].

Gene	Melanoma SNP [%] (Cases)	Colorectal SNP [%] (Cases)	Breast SNP [%] (Cases)	AML SNP [%] (Cases)	CLL SNP [%] (Cases)	T-ALL SNP [%] (Cases)	B-ALL SNP [%] (Cases)
***PIK3CA***	8.16 (7063)	14.16 (24,318)	28.93 (23,448)	0.32 (1567)	0.51 (2344)	1.17 (1367)	0.21 (1456)
***PIK3CB***	4.9 (3426)	2.69 (4878)	2.88 (6212)	0.79 (1399)	0.35 (1738)	0.45 (662)	0.22 (1395)
***PIK3CD***	4.96 (2640)	3.83 (4595)	2.08 (5720)	0.79 (1399)	0.35 (1737)	2.05 (876)	0.5 (1395)
***PIK3CG***	10.82 (3004)	5.62 (4606)	1.74 (5919)	0.78 (1410)	0.17 (1737)	0 (662)	0.07 (1395)
***PIK3R1***	4.25 (4497)	4.59 (5683)	2.6 (9735)	0.79 (1510)	0.41 (1947)	3.44 (1104)	0.53 (1502)
***AKT1***	3.82 (5760)	1.85 (9227)	3.92 (13077)	0.35 (2021)	0.05 (2086)	1.17 (1277)	0.2 (1520)
***AKT2***	4.53 (3624)	1.87 (5020)	1.21 (6190)	0.26 (1512)	0.12 (1737)	0.41 (737)	0.07 (1395)
***AKT3***	4.01 (3537)	3.8 (4947)	4.88 (6251)	0.53 (1512)	1.84 (1737)	0 (654)	0.14 (1395)
***mTOR***	10.93 (3771)	7.37 (5562)	3.36 (8102)	0.99 (1412)	0.69 (1737)	1.82 (991)	0.69 (1453)
***PTEN***	8.1 (5696)	5.23 (10,101)	5.63 (11,038)	0.33 (4191)	0.42 (2122)	12.6 (2336)	0.2 (1527)
***INPP5D***	9.47 (2143)	5.35 (3122)	4.69 (2918)	1.28 (1409)	0.92 (1737)	0.15 (653)	0.29 (1395)
***INPP4B***	4.73 (2750	2.96 (4466)	6.4 (5378)	1.7 (1410)	6.1 (1737)	0.46 (654)	0.65 (1395)
***NRAS***	15.56 (2760)	3.82 (17,606)	0.39 (8160)	13.73 (10,262)	0.66 (2591)	7.81 (1856)	13.11 (3256)
***KRAS***	3.11 (8522)	33.24 (82,451)	1.3 (11,759)	5.12 (7791)	2.06 (2625)	2.71 (1810)	12.6 (3325)
***DUSP6***	1.05 (2185)	1.38 (3122)	0.45 (3092)	0 (1398)	0 (1737)	0 (653)	0.07 (1395)
***PTPN11***	3.79 (4279)	1.83 (6209)	1.25 (7125)	6.1 (7733)	1.68 (1842)	1.2 (1335)	5.34 (2791)
***FLT3***	8.42 (4726)	2.9 (6787)	1.53 (7298)	23.57 (71,050)	0.33 (1811)	3.53 (2123)	5.52 (3318)
***EBF1***	7.37 (2143)	5.09 (3122)	10.56 (2784)	0.85 (1409)	2.99 (1737)	0 (653)	0.5 (1396)
***PAX5***	7.95 (3672)	2.98 (4837)	2.68 (5856)	0.54 (2019)	1.67 (1795)	0.21 (963)	9.17 (2781)
***IKZF1***	8.36 (3122	3.55 (4765)	2.59 (5518)	0.95 (3787)	0.62 (1785)	1.3 (1311)	14.46 (3444)
***TP53***	25.99 (6945)	44.48 (21,508)	26.61 (20,079)	7.16 (6135)	13.28 (4338)	5.04 (1132)	4.15 (2384)

**Table 2:** Overview of biological subtypes of adult and childhood BCP-ALL with INPP5D gene and SHIP1 protein expression (+ low; ++ intermediate; +++ high), according to [106,137].

Subtype	Be Found: Infant	Be Found: Pediatric	Be Found: Adult	SHIP1 RNA	SHIP1 Protein	Trans-Location	Mutation and Deletion	Outcome
**ETV6-RUNX1**		+++		+++	+++	ETV6-RUNX1	KRAS, NRAS, WHSC1, PAX5 del, TBL1XR1 del	favorable
**TCF3-PBX1**		++	+	+	+	TCF3-PBX1	TP53	intermediate
**KMT2A-r**	+++	+	++	+	+	KMT2A fused to AFF1, MLLT1, MLLT3 and other	NRAS, KRAS, FLT3	poor
**Ph-positive**		+	+++	++	+++	BCR-ABL1	PAX5 del, IKZF1 mut + del, CDKN2A/2B del	poor
**Ph-like**		++	+++	++	N/A	CRLF2, ABL, JAK2 EPOR PDGFRB	NRAS, KRAS, JAK2, PTPN11, IKZF1 mut + del, CDKN2A/2B del	poor

## Abbreviations

PtdIns(3,4,5)P_3_, phosphatidylinositol-(3,4,5)-trisphosphate; PtdIns(3,4)P_2_, phosphatidylinositol-(3,4)-bisphosphate; SHIP1, src homology 2 domain-containing inositol phosphatase 1; RAS/ERK, rat sarcoma/extracellular signal-related kinase; GRB2-SOS, growth factor receptor-bound protein 2/son of sevenless; JAK-STAT, Janus kinase/signal transducers and activators of transcription; PI3 kinase, phosphoinositide 3-kinase; AKT, protein kinase B/PKB; mTOR, mechanistic target of rapamycin; PTEN, phosphatase and tensin homolog; AIC, autoimmunity checkpoints; BCR, B cell receptor.
